# Authentic Leadership, Trust (in the Leader), and Flourishing: Does Precariousness Matter?

**DOI:** 10.3389/fpsyg.2022.798759

**Published:** 2022-04-01

**Authors:** Deon J. Kleynhans, Marita M. Heyns, Marius W. Stander, Leon T. de Beer

**Affiliations:** Optentia Research Unit, North-West University, Vanderbijlpark, South Africa

**Keywords:** authentic leadership, trust in the leader, flourishing, precariousness, job overload

## Abstract

**Orientation:**

This study employed a second stage moderated mediation analysis to investigate the influence of authentic leadership on employee flourishing via trust in the leader (mediating variable) and job overload (moderating variable).

**Research Purpose:**

To explore the relationship between authentic leadership and flourishing by considering the indirect effect of trust in the leader as potentially moderated by job overload.

**Motivation for the Study:**

An authentic leadership style, trust in the leader, and job overload may impact employee flourishing. A deeper understanding of the potential interaction effect of trust in the leader and job overload in the relationship between authentic leadership and flourishing may improve individual and organizational productivity.

**Research Approach/Design and Method:**

This study used a quantitative, cross-sectional survey design and PROCESS for moderated mediation. The sample consisted of 314 employees in a prominent steel manufacturing organization in South Africa. The Authentic Leadership Inventory, Workplace Trust Survey (WTS), Flourishing-at-Work Scale, and the Job Demands-Resources Scale were utilized.

**Main Findings:**

The study found that authentic leadership was a significant predictor of flourishing through trust in the leader. Job overload did not moderate the relationship between trust in the leader and employee flourishing.

**Practical/Managerial Implications:**

This study emphasizes the potential role of authentic leadership in fostering a trustful relationship between employees and their leaders. It might result in the increased flourishing of employees. The non-significant influence of job overload on trusting relationships in precarious work contexts was also illuminated.

**Contribution/Value-Add:**

Through the analysis of these relations, organizations may be favorably equipped to optimize the resources required to improve performance. Moreover, the investigation into trust in the leader combined with job overload increases our understanding of supporting and promoting employee flourishing at work.

## Introduction

The turbulent twenty-first century global economic climate has been characterized by organizations plagued with constantly increasing challenges and pressures to meet stakeholder demands (Hameed and Sharma, 2020). To this end, the South African economy is faced with several challenges and weaknesses that include declining investments, decreasing incomes, rising unemployment, and high levels of inequality (Statistics South Africa, 2019). Although South Africa is the most industrialized economy on the African continent, the country’s manufacturing sector, and more specifically, its iron and steel industry is facing a desperate situation as it grapples with the high cost of raw materials, electricity, and reduced market demand, coupled with weak economic growth (Njini, 2019). This situation might lead to workplace-related uncertainty resulting from employees experiencing doubt concerning their future work environment, professional relationships, and job security (Allen et al., 2007). Although being employed during uncertain times may provide income security, it can still result in anxiety and stress (Ererdi et al., 2021) that may affect the employee’s emotional and psychological wellbeing.

More recently, the influence of the coronavirus disease (COVID-19) on the socio-economic and personal environments is unlike anything the world has experienced in the past 75 years (Statistics South Africa, 2020). COVID-19 is recognized as the largest global crisis after the Second World War, and it continues to have a fierce impact on public health and an unparalleled impact on economies and labor markets across the world (International Labour Organization, 2020). The pandemic has created extensive uncertainty that is more impactful than the uncertainty caused by the 2008–2009 financial crisis and comparable to the uncertainty that resulted from the 1929 to 1933 Great Depression (Baker et al., 2020). The pandemic and the subsequent periods of lockdown have had a considerable negative effect on the economy, the work environment, and the wellbeing of individuals (Greyling et al., 2021). To survive, some organizations had to reorganize their operations and resort to downsizing. Downsizing because of restructuring can be described as an event that could result in adapted working conditions (Sonnentag and Frese, 2003) as well as work overload and job insecurity (Foster et al., 2019) and may affect the wellbeing of surviving employees (Shoss, 2017). Sonnentag et al. (2017) state that work overload could lessen an employee’s ability to recover from work strain, resulting in diminished health and wellbeing.

Leadership plays a critical role during challenging and uncertain times (Baran and Woznyj, 2020; Rath et al., 2021) as employees look up to leaders for guidance and direction. A trustful relationship between a manager (leader) and employees is vital as it increases employee commitment and productivity (Krot and Lewicka, 2012). The behavior of leaders during turbulent and unpredictable times can gain or lose followers’ trust and support. Leaders whose words and actions are not aligned will find it hard to be trusted by their team members (Agote et al., 2016). Trust as a key element in the entity’s success is established mainly through the leader’s strategies, plans, and actions (Ndevu, 2019). Authentic leaders can build follower trust via their supportive behavior and the transparent nature of their relationships (Norman et al., 2010). It is thus proposed that a significant positive relationship between authentic leadership (AL) and followers’ trust in the leader (TL) is possible (Agote et al., 2016). Wang et al. (2014) define AL as a genuine, ethical, and transparent leadership style characterized by a positive approach when faced with the challenges related to organizational leadership during times of uncertainty. Rautenbach and Rothmann (2017b) point out that it will be beneficial for organizations to promote AL as it will enhance the flourishing (F) of their employees.

Individuals who flourish are more likely to deal with the vulnerabilities and challenges they are confronted with than are non-flourishers (Schotanus-Dijkstra et al., 2016). The concept of F is represented by hedonic and eudaimonic wellbeing (Huppert and So, 2013). The notion of subjective wellbeing emerged during the 1950s and initially concentrated on hedonic or emotional wellbeing, such as happiness, the balance of positive-negative effects, and life satisfaction (Diener, 1984). Eudaimonic wellbeing incorporates psychological wellbeing and social wellbeing and various components like life purpose, meaning, constructive relationships, and individual development (Salavera et al., 2020). It will be less probable for employees who flourish at work to quit their organization as they are inclined to be above-average achievers, going above and beyond what is expected of them and contributing to organizational performance (Krekel et al., 2019; Redelinghuys et al., 2019). The prevailing uncertain and insecure (precarious) work environment negatively influences employee health and wellbeing (Standing, 2011; Utzet et al., 2020).

Although AL, TL, and F in the workplace have been studied individually, there seems to be a lack of research on the relationship between these constructs within a manufacturing environment and within a precarious context. More specifically, the role of job overload (JO) due to restructuring and how it may moderate the relationship between TL and F is still unclear.

This study intended to determine the nature of the relationship between AL, TL, and F in the work environment, given a context of precariousness (represented by JO in this study). By exploring these associations, companies may be better prepared to apply the internal resources required to achieve improved performance. Moreover, an analysis of TL combined with JO improves our comprehension of possible ways to enhance workplace F.

The job demands-resources (JD-R) model (Demerouti et al., 2001; Bakker and Demerouti, 2017) was established as an excellent research framework (Lesener et al., 2019). When considering the JD-R model, it could be beneficial to have relevant job resources when job demands are high or when an organization, such as a manufacturing entity, is functioning within a challenging context (Bakker et al., 2007). Job demands may involve qualitative demands (e.g., emotional or mental), or quantitative demands (e.g., JO or tempo of change). Job resources may include social resources (e.g., supervisor support), organizational resources (e.g., trust in leadership), and personal resources (e.g., resilience) (Schaufeli, 2017). Occupational wellbeing could increase in conditions where the available job resources enable employees to decrease the potential influence of job demands (Bakker et al., 2014). It could thus be beneficial to identify and utilize available job resources while simultaneously reducing job demands (Lesener et al., 2019). In this study, we will investigate how AL and TL, as job resources, and JO, as a job demand, impact the wellbeing of employees (outcome) in the manufacturing environment.

## Literature Review and Hypotheses

### Authentic Leadership

AL emanates from the attitude and behavior of leaders who have a positive effect on their followers (Rego et al., 2016). The significance of AL in the workplace has been emphasized by literature and practitioners due to authentic leaders being true to themselves, mainly when they display behaviors such as being honest, sincere, and live their values (Leroy et al., 2015). Authentic leaders inspire and motivate subordinates to achieve goals through authenticity and positive moral views, aided by enhanced awareness and effective communication (Crawford et al., 2019). Recent research indicated that AL could have a positive impact on organizations as it has been linked to various outcomes, such as individual creativity, individual performance (Duarte et al., 2021), customer orientation, employee retention (Ribeiro et al., 2020), and employee F (Nair et al., 2021). Although AL has been conceptualized in several ways and evaluated in various empirical research studies (Gardner et al., 2011), possibly the most frequently utilized definition is the one posited by Walumbwa et al. (2008) as “A pattern of leader behavior that draws upon and promotes both positive psychological capacities and a positive ethical climate to foster greater self-awareness, an internalized moral perspective, balanced processing of information, and relational transparency on the part of leaders working with followers, fostering positive self-development” (p. 94).

As depicted in the definition, AL is multidimensional. It consists of balanced processing, self-awareness, relational transparency, and lastly, an internalized moral perspective (Banks et al., 2016). Self-awareness alludes to one’s awareness of one’s strong and weak points in addition to their societal impact (Kernis, 2003). The balanced processing of information relates to the action of leaders who confront their convictions and justly appraise applicable information prior to reaching a conclusion (Walumbwa et al., 2008). An internalized moral perspective pertains to the self-regulated actions of a leader that are steered by internal beliefs and principles rather than behavior directed by external social factors (Hannah et al., 2011). Lastly, relational transparency relates to actions of a leader that demonstrate the leader’s genuine self, their actual convictions, and their emotions to subordinates resulting in reciprocated trust (Wei et al., 2018).

Authentic leaders make ethical decisions and apply balanced processing rather than reaching hasty conclusions. Subordinates may trust the forthcoming actions of a leader because they can utilize prior experiences as an indication of what the future might entail (Clapp-Smith et al., 2009). The behavior and temperament of a leader is an essential factor in the tendency of an employee to trust that leader (Heyns and Rothmann, 2015). When authentic leaders behave with personal consideration and respect toward their subordinates, it will likely enhance the level of trust (Avolio et al., 2004). In support of this view, Hsieh and Wang (2015) suggest that leaders who are perceived to be authentic by subordinates can raise the level of trust among subordinates. This statement was confirmed in recent studies that found that an AL style was favorably linked to TL (Levesque-Côté et al., 2018; Wei et al., 2018; Enwereuzor et al., 2020).

### Trust

Gaining the trust of subordinates is one of the crucial components of being an effective leader. According to the social exchange theory, trust is essential for improving employee and organizational effectiveness (Hsieh and Wang, 2015). Trust is the cement in the relationship that binds employees and their leaders within an organization (Mineo, 2014), leading to organizational outcomes (Dirks and Ferrin, 2002). The trusting relationship employees have with their leader plays a vital role in predicting their workplace experience (Bligh and Kohles, 2013). They may feel that their inputs and ideas matter. Employees’ perception of the trustworthiness of their leader will be influenced by, among others, the following behavioral categories: honesty, sharing of control, and timeous and precise communication while demonstrating empathy (May et al., 2004).

Diedericks and Rothmann (2013) maintain that, based on the social exchange theory, it is plausible that if employees experience a positive relationship with their leader, the relation will cultivate work engagement, emotional wellbeing, and psychological wellbeing. Many of the aspects that could threaten human wellbeing can be ascribed to distrust, as trust is the essence of all relationships (Kleinig, 2018). Flourishing entails the subjective wellbeing of individuals involving emotional, psychological, and social aspects (Keyes and Annas, 2009). Workplace F can be divided into categories of F, mental health, and languishing (Rautenbach and Rothmann, 2017a). Flourishing in the workplace has been described as individuals’ perception that they feel good and function well in their organization (Rautenbach, 2015). Mental health involves the wellbeing of individuals that enables them to develop their abilities, function productively, contribute to their community, and cope with life stresses (Keyes, 2007) in an uncertain environment. Languishing, which is seen as the opposite of F, represents the absence of mental health (Rautenbach and Rothmann, 2017a).

In summary, trust and employee wellbeing are not only desirable in themselves but are also valuable in improving the recruitment, performance, and retention of employees (Krekel et al., 2019). Given this, it is possible to argue that TL will influence employee F in the work and organizational context (Pillai et al., 1999; Kelloway et al., 2012).

### Precariousness

One of the challenges confronting organizations is the decline in the conventional long-term occupational relational obligation, mutual exchange, and employment security, as these have been substituted by elevated precariousness (Utzet et al., 2020). The change in working conditions, and particularly work overload, contribute to the occupational stress experienced by employees and may be negatively associated with their wellbeing (Laurence et al., 2016). Although other reasons exist, overload may occur due to the new legal or market requirements, the implementation of new technologies, staff reductions and reorganization (García-Arroyo and Osca, 2019). Morgan and Zeffane (2003) analyzed the influence of different organizational changes, such as technological, structural, and work roles, on employee trust in leaders. Their study indicated that the uncertainty and insecurity due to reorganization and change significantly eroded TL. The negative emotions of employees toward ongoing changes and uncertainty result in reduced levels of trust in the organization, senior management, and line management (Kiefer, 2005). The uncertainty and vulnerability brought about by ownership and top management changes, strategic reorientation, and significant organizational restructuring will challenge and weaken employees’ TL (Sørensen et al., 2011).

It may also be argued that the measure of trust of employees in their leader could have a buffering consequence, as when employees have belief in their leader’s judgment and good intentions, they may experience less uncertainty during times of change (Gundhus, 2018). Since trust can serve as lubrication for social systems, it may imply that employees can rely on the promises of others within their organization (Martins and von der Ohe, 2011). Werbel and Henriques (2009) state that trust is “the willingness to make oneself vulnerable to another person despite uncertainty regarding motive and prospective actions” (p. 781), implying that peril, exposure, and doubt are part of trustful relationships (Heyns and Rothmann, 2015). Another element of trust is the expectation that the future actions of another party will be beneficial or at least not harmful to one’s interests (Cheng et al., 2019). Most researchers describe trust as being vulnerable and exposed to another party due to positive future expectations. The trust employees have in their leaders and their employer influences the interpretation of the potential threat of insecurity and uncertainty resulting from change and reorganization within an entity (Arnold and Staffelbach, 2012). As a result, employees might perceive the situation as less threatening because they believe in their organization’s ability, benevolence, and integrity (Arnold and Staffelbach, 2012).

The perception of followers regarding the interaction and relationship with their leader can be a form of support and a resource that can affect their wellbeing (Halbesleben et al., 2014). For example, follower-leader trust represents the employee’s perception of communicating openly with the leader regarding job-related challenges without fear of negative consequences (Fulk et al., 1985). The mentioned characteristic of TL has been linked to follower wellbeing (Liu et al., 2010; Braun et al., 2013). Russell (2008) describes subjective wellbeing as comprising emotional wellbeing and positive functioning (psychological and social wellbeing). When employees trust their leader, it will beneficially influence their psychological wellbeing, reducing their perceived level of risk and vulnerability (Kelloway et al., 2012). Trust in the leader favorably impacts on the social wellbeing of laborers because the trusting relationship will lead to a sound social bond between them (Pillai et al., 1999). When employees trust their leader, it will influence their emotional wellbeing positively. They do not have to consume emotional and cognitive energy and resources while attempting to safeguard themselves from their leader due to low levels of trust (Kelloway et al., 2012).

The information above may suggest that the level of TL is associated with F in the workplace. A precarious context might negatively influence the relationship between TL and employee F. Precariousness in this study is represented by job/work overload resulting from organizational restructuring and staff reduction. The mentioned changes might imply that employees receive more responsibilities and work longer hours, which may impact negatively on their wellbeing.

### Flourishing

Organizations are becoming increasingly aware of promoting and sustaining employee wellbeing to maintain their competitive advantage (Nielsen et al., 2017). Keyes (2005) describes F as “a syndrome of subjective wellbeing which combines feeling good (emotional wellbeing) with positive functioning (psychological and social wellbeing)” (p. 7). Although individuals who flourish are those who experience both high levels of eudaimonic and hedonic wellbeing, not many researchers focused on both domains when studying flourishing (Schotanus-Dijkstra et al., 2016). Ho and Chan (2022) describe F in the context of perceived organizational support according to the definition of Diener et al. (2010), who state that F points to psychosocial performance, which is accomplished through the satisfaction of the human psychological desires for meaning, human bonds, self-esteem, proficiency, and purpose. When individuals flourish in their workplace, they feel good (i.e., they experience job satisfaction and positive emotions at work), perform psychologically well (i.e., they are dedicated, energetic, determined, and find meaning and purpose at work), and function effectively from a social perspective (in aspects, such as social acceptance, social coherence, social contribution, social integration, and social growth) (Rothmann, 2013). The significance of wellbeing in the workplace is evident from related positive outcomes, such as work satisfaction, health, and job performance (Grant and Campbell, 2007). The many changes in the global economy and the work environment have made the occupational situation more insecure and uncertain, resulting in the increased perceived precariousness of employees and have put their sustainable wellbeing at risk (Giunchi et al., 2019). The era we live in is increasingly characterized by precarious employment, representing a fundamental shift toward widespread uncertainty and insecurity, potentially leading to employees experiencing low levels of subjective wellbeing (Kalleberg, 2018). In the complex and ambiguous work environment, job insecurity has become a significant stressor with adverse effects on employees’ wellbeing (De Witte et al., 2016; Jiang and Lavaysse, 2018).

The modern organizational setting is increasingly typified by complex work demands, work overload (Vogel, 2012) and longer working hours, given a 24/7 operational process in most organizations (Barnett and Hyde, 2001). Considering the JD-R model framework (Bakker and Demerouti, 2017), JO is seen as one of the job demands that may affect employee wellbeing negatively. Janse van Rensburg and Rothmann (2020) state that job demands represent a job’s psychological, tangible, societal, or organization-related attributes requiring actual and intellectual effort and are associated with specific physical and intellectual expense. Job demands may be synonymous with, among others, overtime work (Richardsen et al., 2006), job insecurity and time-associated demands at the workplace (Mauno et al., 2007), and challenges resulting from reorganization and workload (Bakker et al., 2003).

### The Focus of the Present Study

The present research investigated the relation between AL, TL, F, and precariousness (reflected by JO).

Supervisors who display authentic behavior by demonstrating mindfulness, transparency in their relations with others, a sound moral point of view, and a well-balanced approach to reaching conclusions will most likely establish a trusting relationship with their followers (Hassan and Ahmed, 2011). This could be related to the leader’s accommodative and considerate behavior and open and transparent communication (Clapp-Smith et al., 2009). Additionally, authentic leaders promote subordinate TL due to leadership authenticities, such as relational transparency and authentic behavior that are positively associated with the subordinate’s TL (Hassan and Ahmed, 2011). Authentic leadership was found to be a principal job resource viewed from a JD-R point of view. It assists in establishing a positive and healthy work environment, resulting in numerous beneficial work-related outcomes (Adil et al., 2019).

Liu et al. (2010) and Helliwell (2011) established that the level of perceived trust directly impacts on the subjective wellbeing of individuals. Flourishing points to personal subjective wellbeing, which focuses on how employees evaluate their experiences within different contexts (Janse van Rensburg et al., 2017). Employees who trust their leader reportedly experience a sense of security and comfort because they feel their leader has their best interest at heart; therefore, they feel less exposed to being harmed by their leader (Liu et al., 2010; Kelloway et al., 2012). When the sense of uncertainty and vulnerability is reduced through TL, it affects employee wellbeing (Kelloway et al., 2012). Prior studies have found that TL can mediate the link between AL, as a predictor, and organizational citizenship behavior and work engagement (Altuntas and Baykal, 2010; Hsieh and Wang, 2015). However, when employees are of the opinion that they cannot trust their leaders, they may feel anxious, adversely affecting their wellbeing (Dirks and Ferrin, 2002). One can assume that the distress will be more severe in uncertain times.

The global economic and technological changes have brought about increased employment uncertainty in many sectors of the economy and have led to a rise in precariousness (Standing, 2014). Job overload in the form of additional work and longer working hours may result from changes in the working environment. Demerouti et al. (2009) found that employees who were confronted with many job demands, such as workload and physical demands, reported reduced wellbeing.

Uncertainty and insecurity created in an organization will almost certainly threaten the trust relationships between employees and their leaders (Sørensen et al., 2011). Although trust is highly desirable, organizations find it challenging to develop interpersonal trust and TL due to a highly uncertain and insecure business environment (Zanini et al., 2009). Because employment is a source of meaning, identity, and personal connection, employment-related uncertainty and instability might threaten the subjective follower wellbeing (Blustein, 2006).

The JD-R model suggests that when faced with stressful conditions (JO), employees will be likely to draw on their resources (Demerouti and Bakker, 2011), such as the trust in their leaders (Visser et al., 2016) and the perceived AL behavior of these leaders (Adil et al., 2019).

Based on the information above, it is viable to argue that although AL will affect the trust relationship between employees and their leaders, the level of trust will determine the influence a precarious work context, characterized by JO, will have on the F of employees.

**FIGURE 1:**
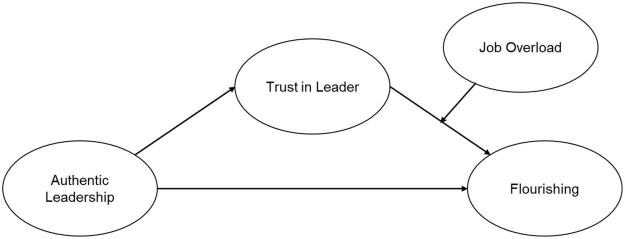
The conceptual model.

Based on the discussion as set out above and the conceptual research model (Figure 1) as reference, the following hypotheses were formulated:

Hypothesis 1: Authentic leadership is positively associated with trust in the leader and flourishing.

Hypothesis 2: Authentic leadership is positively related to flourishing through trust in the leader.

Hypothesis 3: The indirect effect of authentic leadership on flourishing through trust in the leader is moderated by job overload.

## Materials and Methods

### Participants

The target population for this study included the managers on different levels employed at the various plant sites of a noteworthy manufacturing organization in South Africa. Questionnaires were distributed to 570 potential participants, and 314 completed questionnaires were received back, resulting in a 55% response rate. Stratified random sampling was utilized during data collection.

The attributes of the respondents: Of the respondents 12.4% indicated that they were between 31 and 40 years of age. A total of 13% reported that they were employed at senior managerial level, and 30.5% indicated that they had 31–40 years of service. Of the target group, 63.3% stated that they had 0–10 years of experience in their present job. Lastly, 22% of the participants said that they were employed in the corporate services environment.

### Measures

Respondents were requested to fill out a biographical information form and the survey for appraising AL, TL, F, and JO.

To measure employee perception of AL characteristics, the Authentic Leadership Inventory (ALI; Neider and Schriesheim, 2011) was utilized. The ALI comprises four dimensions (self-awareness, internal moral perspective, balanced processing, and relational transparency) that are measured using 14 items. Examples of the items include: “My leader openly shares information with others” and “My leader encourages others to voice opposing points of view.” A five-point Likert-type scale ranging from 1 (strongly disagree) to 5 (strongly agree) was utilized to score the individual items. Previous studies established admissible reliabilities with Cronbach’s alpha coefficients varying between 0.74 and 0.90 (Neider and Schriesheim, 2011; Men and Stacks, 2014). The ALI was also confirmed as reliable in a South African context (α = 0.93; Stander et al., 2015).

Trust in the leader was measured by utilizing one scale of the Workplace Trust Survey (WTS) comprising nine items (Ferres and Travaglione, 2003). Items were scored by applying a seven-point Likert-type scale ranging from 1 (strongly disagree) to 7 (strongly agree). Example items include: “I proceed on the basis that my supervisor will act in good faith” and “I feel that my supervisor is available when needed” (Ferres and Travaglione, 2003). Cronbach’s alpha reliability coefficients between 0.90 and 0.97 were reported during studies conducted in South Africa (Ferres and Travaglione, 2003).

The eight-item JO portion of the Job Demands-Resources Scale, established by Jackson and Rothmann (2005) was applied to assess work overload. Mental and emotional load and items relating to pace and amount of work are included. Item examples are: “Do you have too much work to do” and “Does your work put you in emotionally upsetting situations.” A scale ranging from 1 (never) to 4 (always) was utilized to rate the questions. The Cronbach’s alpha reliability coefficient was found to be 0.75 (Rothmann et al., 2006).

Workplace F was measured by applying the Flourishing-at-Work Scale Short Form (FAWS-SF) which includes 17 items (Rautenbach and Rothmann, 2017a). These items were assessed by applying a six-point scale that varies from 1 (never) to 6 (every day). Emotional wellbeing was measured by three items (e.g., “During the past month at work, how often did you experience satisfaction with your job?”), psychological wellbeing by nine items (e.g., “During the past month at work, how often did you feel your work is meaningful?”) and social wellbeing by five items (e.g., “During the past month at work, how often did you feel you had something important to contribute to your organization?”). The validity and reliability of the FAWS-SF was tested under South African conditions and found to be valid and reliable (α > 0.70) (Rautenbach and Rothmann, 2017a).

Single method bias is regularly mentioned as a survey design limitation. This was, however, mitigated by the limitation of the number of items contained in the research questionnaire and by utilizing alternative response formats (Podsakoff et al., 2012). Additionally, the respective questions were worded in a clear and concise manner (Podsakoff et al., 2003). Moreover, respondents were reassured of their anonymity with no risk for their employment—this reassurance contributed to genuine responses (Steenkamp et al., 2010). Lastly, standardized questionnaires were administered.

### Statistical Analysis

For statistical analysis, the software program R version 3.5.3 was utilized in RStudio (RStudio Team, 2020). The best-fitting measurement model was identified by conducting a confirmatory factor analysis (CFA) with the “lavaan” package (Rosseel, 2012). Structural equation modeling (SEM) was carried out to evaluate the research model and determine how the data fit the models. The items contained in the questionnaires were dealt with as continuous data. The maximum likelihood with robust standard errors estimator (MLR), known for its robustness to the abnormality of input data (Wang and Wang, 2012), was applied to estimate the model on the data with no missing values.

To pinpoint the best-fit model, the following fit indices were utilized. Absolute fit was evaluated by determining the chi-square value. The comparative fit index (CFI) and the Tucker-Lewis index (TLI) were used as the incremental fit statistics. The cut-off value for the CFI and TLI fit indices was 0.90 (Wang and Wang, 2020). The root mean square error of approximation (RMSEA) and standardized root mean square residual (SRMR) representative of the alternative fit indexes were calculated and considered. RMSEA and SRMR values below 0.08 were seen as indicative of a good fit between the model and the data (Wang and Wang, 2012).

The objective of this study was to determine the conditional indirect effect of AL on F via TL at different values of JO (moderation analysis). The research consisted of the association between AL and TL (a-path), TL and F (b-path), the direct influence of AL on F (c-path) and lastly, the interaction consequence of TL and JO on F. This conditional indirect effect was tested using PROCESS Model 14 (Hayes, 2013) to ascertain whether second stage moderated mediation was evident. Specifically, the latent variable factor values from the CFA model were administered as a new data set as an input to PROCESS as it cannot estimate latent variables itself. Bootstrapped confidence intervals were generated to calculate the index of moderated mediation (IMM). Zero inclusion in the lower and upper confidence intervals pointed to a meaningful conditional indirect effect (Hayes, 2015). Composite reliability was calculated using the sum of squares of the standardized loadings and variance of error terms (Raykov, 2009). The cut-off value of 0.70 was viewed as admissible (Wang and Wang, 2012).

## Results

### Confirmatory Factor Analysis

A measurement model was specified with AL as a second-order factor, indicated by the four first-order components of AL that in turn were indicated by the respective items for each of the four components. Flourishing was also treated as a second-order factor, while TL and JO were measured by observed items as they do not consist of individual components. The chi-square test was significant (*p* < 0.01), suggesting a less than perfect fit to the data (χ^2^ = 1813.47, df = 1067, *p* < 0.01). However, this fit measure is known to be oversensitive to sample size, and researchers should report multiple fit indices (Hancock and Mueller, 2010). The respective alternative fit indices pointed to an approximate fit to the data: The TLI (0.903) and CFI (0.907) values were higher than the 0.90 cut-off point, the RMSEA value suggested a good fit (0.047), and SRMR indicated a good fit (0.069). Due to Model 1 closely resembling theory, it was identified as the best model.

Table 1 reflects the correlation matrix with descriptive statistics (e.g., means and standard deviations) and the rho reliability coefficients of Raykov (2009). As summarized by the authors, the results in Table 1 indicate that Raykov’s rho coefficients were well above the minimum threshold and considered reliable as the values were more than 0.70.

**TABLE 1:** Correlation matrix, including means, standard deviations, and reliabilities.

Variable	*M*	*SD*	ρ	1	2	3
1. AL	3.61	0.65	0.98	−	−	−
2. TL	5.25	1.22	0.96	0.82^‡**^	−	−
3. JO	3.18	0.38	0.71	0.09	0.03	−
4. F	4.45	0.77	0.93	0.43^†**^	0.54^‡**^	–0.08

M, mean; SD, standard deviation; ρ, composite reliability coefficient; ^†^r > 0.30; ^‡^r > 0.50; **p < 0.01.

Variables were conceptualized as X = AL, M = TL, W = JO, Y = F; and as depicted in Table 1, showed mixed results. Authentic leadership showed positive, statistically significant associations with TL (*r* = 0.82, large effect) and with F (*r* = 0.43, medium effect). This outcome provides support for Hypothesis 1. However, TL was not notably associated with JO (*r* = 0.03, *p* > 0.05) but indicated a statistically strong positive association with F (*r* = 0.54, large effect). Lastly, JO was negatively but not statistically significantly associated with F (*r* = −0.08, *p* > 0.05).

### Testing Interaction Effects

The Hayes (2017) mediation, moderation, and conditional process analysis technique was applied, using PROCESS Model 14. The moderated mediation analysis consisted of the testing of association between AL (X) and TL (M, a-path), TL and the outcome variable F (b-path), the direct effect of AL on F (c’-path) and, lastly, the interaction effect of TL and JO on F (M*W).

The moderated mediation assessment was non-significant: β = 0.08; SE = 0.24; CI (−0.41; 0.52), and the bootstrapped estimates indicated a non-significant moderated mediation effect because zero did span the CI.

This outcome suggests that the direct and indirect influences, in addition to the conditional indirect influences, were similar when JO was added as moderator (Hayes, 2018). Additionally, the second stage moderated mediation model explained 40% of the variation in F when TL was utilized.

The conditional indirect effects are displayed in Table 2.

**TABLE 2:** Research model: moderated mediation results.

(2nd Stage)	β	SE	*t*	*p*	95% CI (Lower)	95% CI (Upper)
AL → TL (a)	1.93	0.07	29.36	0.001*	1.80	2.06
TL → F (b)	0.47	0.05	8.78	0.001*	0.36	0.57
AL → F (c’)	–0.22	0.12	–1.86	0.063	–0.46	0.01
TL*JO → F (c-c’)	0.04	0.11	0.36	0.714	–0.18	0.27
**Indirect effects and IMM**						
JO—Low (−0.23)	0.88	0.13			0.62	1.14
JO—Moderate (0.01)	0.90	0.13			0.65	1.15
JO—High (0.26)	0.93	0.15			0.62	1.20
IMM	0.08	0.24			–0.41	0.52

β, unstandardized beta coefficient (PROCESS does not provide standardized in moderation results); SE, standard error; *p ≤ 0.001.

The only direct effects in the model that were significant were the regressions from AL to TL (β = 1.93; SE = 0.07; CI [1.80, 2.06]) and TL to F (β = 0.47; SE = 0.05; CI [0.36, 0.57]). The direct regression from AL to F (β = −0.22; SE = 0.12; CI [−0.46; 0.01]) and the interaction effect were insignificant (β = 0.04; SE = 0.11; CI [−0.18, 0.27]). Even though there was no significant moderated mediation, evidence existed that only the indirect effect from AL to F through TL was meaningful as all the values (even at different levels of JO) were similar and did not include zero. Therefore, there was only evidence of an indirect effect but no moderation of that effect. Support for Hypothesis 2 and Hypothesis 3 was hereby suggested.

## Discussion

The objective of this study was to position AL and TL (job resources) as facilitators of F. The process TL through which AL influences F was also analyzed. Lastly, the study determined whether a contextual factor, such as JO, will influence the association between AL, TL, and F. To our knowledge, no study involving the constructs of AL, TL, JO, and F has been conducted before.

The results underpin the significant and influential part AL can play in the manufacturing industry. Firstly, the study indicated an affirmative connection between AL and TL, thereby supporting the first part of Hypothesis 1 (AL is positively associated with TL). This outcome implies that increased levels of AL may result in higher TL and employee F. This finding supports the suggestion that AL functions as a job resource as stipulated by the JD-R model (Lee et al., 2020). Similar results came from previous research studies (Zhang et al., 2018; Adil and Kamal, 2019; Álvarez et al., 2019). The behavior and character of a leader may influence the tendency of an employee to trust that leader (Heyns and Rothmann, 2015), especially when leaders keep their promises (Wang and Hsieh, 2013). The trusting relationship between employees and leaders can also result from authentic leaders acting transparently when dealing with employees (McAuliffe et al., 2019), giving them hope to cope with the future uncertainty. During challenging times (e.g., restructuring and the COVID-19 pandemic), employees must trust their leader to implement initiatives that may mitigate the negative effect of these challenges on the organization and followers. This result also implies that leaders have a positive influence on employees when they listen to them and consider their opinions and ideas while being transparent in their dealings with them as these employees will feel confident and determined to perform at their best. It can be expected that a leader who exhibits these behavioral characteristics will promote feelings of trust in followers because they will feel comfortable to share information with the leader, test ideas, and improve their work engagement levels.

Secondly, the relationship between AL and F was established, which confirms the second part of Hypothesis 1 (AL is positively associated with F). This outcome is similar to the results of research which established that authentic behavior has a profound influence on the wellbeing of employees (Ilies et al., 2005; Rautenbach and Rothmann, 2017b; Kerns, 2018). Authentic leader behavior also promotes organizational citizenship behavior, follower creativity, and employee performance (Ribeiro et al., 2018). According to Kernis (2003), authentic leaders are positive and optimistic, and these positive emotions tend to spill over to employees as if these emotions were contagious. The positive environment created by authentic leaders is preserved by a mutual emotional transfer that strengthens subordinate wellbeing (Rautenbach and Rothmann, 2017b).

Thirdly, even though the IMM was not found to be meaningful as expected, the results did support an indirect link joining AL and F through TL, providing support for Hypothesis 2. Authentic leaders can foster a climate of inclusion, trust and support that influences employee behavior and attitudes. In an environment where information is shared openly and freely, and employees are encouraged to provide inputs and develop themselves, the leader-follower relationship will benefit. It is highly likely that when followers have a positive relationship with their leader, employee wellbeing and engagement will be promoted (Diedericks and Rothmann, 2013). The relation linking AL and F may be enhanced when TL is elevated rather than reduced, as TL will function as a supplementary job resource that may increase leadership influence.

Lastly, the indirect association of AL with F via TL was not found to be affected by a moderating effect of JO on employee F—a strong association between TL and employee F was not moderated by the presence of JO, resulting in Hypothesis 3 not being accepted. The study results suggest that a fluctuation in the level of JO did not influence the direction or size of the association between TL and F. This outcome was unexpected as many organizations (including the organization used in this study) had to revert to restructuring of their operations involving downsizing that could have resulted in heightened work demands and the overload of remaining employees (survivors) (Kozlowski et al., 1993). Downsizing may also result in the remaining employees experiencing feelings of job insecurity, anger, depression, decreased motivation, and morale that could have a negative effect on their wellbeing (Kets de Vries and Balazs, 1997).

Given that the target entity had to undergo restructuring of its operations before, it is likely that the effect of possible JO was mitigated by how the leaders dealt with these initiatives in the past (Penger and Černe, 2014; Heyns and Rothmann, 2015). The finding might be explained by the authenticity demonstrated by the leaders through leading by example while exhibiting optimism, hope and confidence (Gardner et al., 2005). These positive attitudes could have a trickle-down effect that could lead to the same positive emotions among employees (Frederickson, 2003). Furthermore, when making decisions, such as the redistribution of work after a downsizing exercise, authentic leaders are likely to assess all relevant information objectively and fairly before reaching a final decision. Employees working under these conditions are encouraged to support and assist one another (Walumbwa et al., 2010) and make one another aware of the benefit such behavior might hold for all role-players involved (Brown et al., 2005). This type of behavior, coupled with the transparent and frequent sharing of relevant information, may lessen the possible negative effect JO could have on employees and their wellbeing. Lastly, the fact that the managers (leaders), who were the target population of this research, underwent skills training to equip them to handle the effects of JO brought about by restructuring initiatives might have contributed to lessen the impact of JO on the relation between the trust they had in their leader and their wellbeing. Among others, the training included the prioritization of work, efficient time management, learning to delegate tasks, developing the confidence to say “no” to additional work when warranted, using technology to their advantage, practicing relaxation techniques, and reaching out to their team leader when feeling overwhelmed by the allocated work.

Hence it is evident that manufacturing organizations should consider promoting and developing AL among their leaders as it could instill TL in employees and possibly enhance employees’ psychological, emotional, and social wellbeing. Even though the manufacturing entity where the study was conducted was confronted with uncertainty and volatility due to restructuring when the study was carried out, the results suggest that despite operating in a turbulent context, promoting an AL style can have beneficial individual consequences. In uncertain times, leaders should admit and embrace their discomfort (self-awareness and transparency), support employees to understand the prevailing complexities (balanced processing), involve and value employees’ input (relational transparency), and clarify the bigger picture and purpose (internal moral perspective) while managing ambiguity.

The identified research gap was addressed by confirming the association between AL, TL, and F. This study also contributed to theory by suggesting that JO as a type of precariousness in a business context does not moderate the relation between TL and employee F. The positive association of AL with TL and employee F highlights the potential value of AL in a broader South African context and the manufacturing industry. The findings of this study may also be extended to other industries that find themselves in a precarious business environment and that depend on the manufacturing industry for economic advancement. While comprehending the mechanisms of AL’s boundary conditions are essential for theory development, mediated-moderation research related to AL is limited. This study attempted to add value by clarifying and understanding the leader-follower influencing process by eliminating some boundary conditions.

### Managerial Implications

Leaders should enable employees to identify what is within their control and what threats they must accept. External threats are beyond their control, but leadership development and creating a trustful climate are within the organization’s control.

The outcome of this study suggests three approaches that organizations can follow to enhance the F of their employees through the development of AL and strengthening TL. The first suggested approach is to incorporate interventions to increase AL. This can be achieved by implementing an organizational culture that not only promotes authenticity but also encourages leaders to undertake a journey of self-awareness to discover their strengths, limitations, and emotions while being true to themselves and others.

The second recommended approach is to introduce interventions that will encourage leaders to be more transparent, sincere, supportive, and true to their word while enhancing TL, which will potentially result in increased employee wellbeing. Additionally, when trusted leaders encourage frequent open and honest two-way communication opportunities and provide honest feedback and recognition while demonstrating caring and empathy, it may affect the F of their followers. One way to install a two-way interactional approach is to develop leaders as coaches. Not only will it enhance self-awareness within the leader, but it will also reinforce focusing on strengths, continuous feedback, and a development culture.

Thirdly, involving employees to rethink jobs and allow creative job crafting can reduce the load and enhance meaningful work experiences.

### Limitations of the Study and Recommendations for Future Studies

This research was not without limitations. First, self-report measures can cause relationship over-inflation and bias (Podsakoff et al., 2003). However, since the personal opinions of respondents were of interest, self-report measures were still considered the best way to obtain the respondents’ subjective views (Spector, 2019). An effort was nevertheless made to limit possible biased reporting by the way in which the questionnaires were constructed, as was discussed earlier on in this article. A concerted effort was also made to ensure trustworthy responses. For instance, the respondents were assured that participation was voluntary, that there were no right or wrong answers, and that the data would only be used in an aggregated format so that there would be no means to trace inputs back to the individual level (Steenkamp et al., 2010).

The present study investigated the relationship between variables in a combination that was not studied before; therefore, a cross-sectional design was considered appropriate (Spector, 2019). The researchers also acknowledged the view of Spector (2006, 2019) that suggest that currently, no measure exist in the literature that could comprehensively test for CMV resulting in it being completely ruled out. Due to the chosen research design, generalizability is unfortunately limited and causal inferences involving the variables cannot be made.

Third, the study focused on only one manufacturing company, and it may thus be interesting to determine whether the same results would be achieved when including more than one manufacturing entity.

Future studies could explore how different categories of resources impact the processes through which AL associates with follower outcomes. Although the current study focused on the respective management levels within the targeted manufacturing organization, future studies could include employees employed in non-supervisory roles and those on the lower levels of the entity. Similar future studies should consider a longitudinal approach to interrogate the relation between AL, TL, and F over time to determine whether changes in the business context will affect the findings of this study. Adding organizational constructs, for instance work engagement, psychological safety, and organizational citizenship behavior, with AL and TL as the main focus, could also form part of future studies.

## Conclusion

The study’s results underpin the significant and influential part AL can play in the manufacturing setting. When leaders display an internalized moral perspective, self-awareness, relational transparency, and balanced processing in the workplace, they are likely to enhance employee trust. Manufacturing organizations should consider promoting and developing AL among their leaders and managers to instill TL with the possibility of enhancing employees’ psychological, emotional, and social wellbeing. The findings suggest that JO did not moderate the association between TL and F. Through TL, the behavior of authentic leaders influences the achievement of F and potentially increases employee performance. Lastly, although at the time the study was conducted, the manufacturing company that took part in this study was confronted with uncertainty and volatility due to restructuring, the research findings indicate that an AL style could result in favorable individual effects notwithstanding the turbulent context.

## Data Availability Statement

The raw data supporting the conclusions of this article will be made available by the authors, without undue reservation.

## Ethics Statement

The study involving human participants were reviewed and approved by the Economic and Management Sciences Research Ethics Committee (EMS-REC) of the NWU Potchefstroom South Africa. Ethics number (NWU-00609-20-A4). The patients/participants provided their written informed consent to participate in this study.

## Author Contributions

DJK was the first author. MMH and MWS made contributions regarding the conceptualization, review, and editing of the study. LTdB performed the statistical analysis. All authors contributed to the article and approved the submitted version.

## Conflict of Interest

The authors declare that the research was conducted in the absence of any commercial or financial relationships that could be construed as a potential conflict of interest.

## Publisher’s Note

All claims expressed in this article are solely those of the authors and do not necessarily represent those of their affiliated organizations, or those of the publisher, the editors and the reviewers. Any product that may be evaluated in this article, or claim that may be made by its manufacturer, is not guaranteed or endorsed by the publisher.
